# Clinical association between teeth malocclusions, wrong posture and ocular convergence disorders: an epidemiological investigation on primary school children

**DOI:** 10.1186/1471-2431-13-12

**Published:** 2013-01-23

**Authors:** Armando Silvestrini-Biavati, Marco Migliorati, Eleonora Demarziani, Simona Tecco, Piero Silvestrini-Biavati, Antonella Polimeni, Matteo Saccucci

**Affiliations:** 1 Department of Orthodontics, University of Genova, Genoa, Italy; 2 Dental Practitioner, Savona, Italy; 3 Department of Medical, Oral and Biotechnological Sciences, University G. D’Annunzio Chieti, Chieti, Italy; 4 Dental Practitioner, Postural Gnathologist, Genoa, Italy; 5 Department of Pedodontics, Sapienza University of Rome, Rome, Italy

**Keywords:** Teeth malocclusions, Wrong posture, Ocular convergence disorders

## Abstract

**Background:**

As the various systems in the body are inter-connected to form a single structural unit, a pathological condition in one area can also affect other areas. There are many known correlations between the visual and motor system. The importance of visual function, particularly the paracentral peripheral field of view, in motor coordination, ambulation and the maintenance of balance has been amply demonstrated.

In line with current medical principles, which are moving towards a more holistic view of the human body, this study aims to investigate, in an interdisciplinary manner, the incidence of dental malocclusions together with posture and eye convergence disorders.

**Methods:**

Six hundred and five children attending at the 3^rd^, 4^th^ and 5^th^ years of seven Genoa primary schools were examined. Each child underwent the following examinations: (i) dental/occlusal; (ii) orthoptic; and (iii) postural. Occlusal data concerned the presence of cross-bite, midline deviation with a mandibular shift, bad habits and deep or open bite.

Postural assessment involved frontal and lateral inspection, investigation during trunk flexion and ambulation, and note of any asymmetry in the lower limbs. The recorded orthoptic data included those pertaining to ocular dominance, a cover test, convergence and the Brock string test.

**Results:**

A prevalence of cases with an unphysiological gait was found in patients with overjet (14.70%) or overbite (14.87%), while the percentage of patients with normal occlusion that showed an unphysiological gait was 13.08%. Also, about 93.8%–94.2% of children showed normal legs without dysmetry, with no difference in respect to the type of occlusion. Subjects with an open bite or deep bite showed a slightly different distribution of right or left dominant eyes.

**Conclusion:**

About 13% of children showed a pathological gait and, among them, vertical anomalies of occlusion (deep bite or open bite) were prevalent with respect to the other occlusal defects. The vertical dimension of occlusion revealed a slight relationship with the proper dominant eye. Postural, orthoptic, osteopathic and occlusal variables were often clinically associated, and therefore these disorders appear to request a multidisciplinary medical approach for their treatment.

## Background

As the various systems in the body are inter-connected to form a single structural unit, a pathological condition in one area can also affect other areas. In particular, the skeletal muscles play a decisive role in the coincidence of various disorders, because of the continuous anatomical and functional “chain” they form between the skull, lower jaw, spine, limbs and pelvis [1-5].

Indeed, if a situation of muscular high tension arises in one of the links in this chain (mandible, hyoid, vertebrae, pelvis and limbs), it is immediately transmitted to the rest of the body. As a consequence, the body loses its state of equilibrium, giving rise to compensation mechanisms, for example muscular tension in other antagonistic parts of the body. In this way, dental malocclusion can be associated with misalignment of the mandible, one of the links in the muscular chain, leading to hyper-contraction of the masticatory muscles [6]. This tension forces the rest of the body to react, imposing postural modifications brought about by the contraction of other muscles in the chain. In fact, the incidence of malocclusion in orthopedic patients reported in the literature ranges from 83% to 87% [7]. Furthermore, it is now widely recognized that problems involving the stomatognathic apparatus and alterations in feet posture can cause kinetic dysfunction, leading to pathologies in the ascending and descending spinal tracts [7,8].

Perinetti et al. [9] investigated malocclusal traits correlated with body posture alterations. A limited number of significant correlations were observed, mainly for overbite, when using multivariate models.

Moreover, it has become increasingly evident that disorders of the sensory nervous system can have a considerable influence on regulation of motor function [8]. In fact, modifications in these sensory neurons can cause parafunctional alterations and pathologies in apparently unrelated body districts, For instance, dental malocclusion can cause pain elsewhere in the body. Because of its position, misalignment of the mandible can also cause the position of the pupillary line to be momentarily altered, provoking the intervention of ocular muscles to keep the gaze straight.

There are many known correlations between the visual and motor systems, and the importance of visual function, particularly the paracentral peripheral field of view, in motor coordination, ambulation and the maintenance of balance has been amply demonstrated [10]. In fact, to follow an object in motion, the eye needs to be able to coordinate the movement of the head and neck. The musculature controlling eye movement is closely connected to the stomatognathic system. Fibers emerging from the muscle spindles and palisade endings in the oculomotor muscles, especially the lateral rectus muscle, form pathways to the oculomotor nuclei and the trigeminal nucleus [11-13]. Ocular defects that can be linked to dental malocclusion are convergence defects, heterophoria, heterotrophia and esodeviations.

This study is based on three presuppositions: 1) a mandibular shift may cause a positional head adaptation [13]; 2) ocular phorias may cause a head compensation posture called ocular torticollis [14]; and 3) a head compensation posture causes body adaptation positions, to maintain a center of gravity compatible with the upright position [15]. Head positions enhance postural compensation phenomena; thereby head position is a key point in body balance. This is demonstrated by the great incidence of cervical pain due to muscular tension, which means head posture controls hyperfunctions [16,17].

In this study, the aim was to determine if these disorders occur with significant frequency in children (aged 7–10 years). We analyzed *in primis* dental malocclusions (by an orthodontist) and subsequently orthophoria was recorded (by an orthoptist). Then the cranio-sacral respiratory rhythm was tested (by an osteopath). The incidence and concomitant frequency of these disorders were statistically analyzed to understand whether a causal connection existed in situations where patients were forced to carry out functional compensations in different body districts.

## Methods

In this epidemiological study, conducted with the collaboration of the local health authority (ASL 3, 1^st^ District, School Medicine Services), 605 children (mean age 8.5 ± 2.3 years; 45% males; 55% females) attending the 3^rd^, 4^th^ and 5^th^ years of seven Genoa primary schools were examined.

Before any clinical research was performed, the parents of the children attending the selected classes were asked to provide informed consent and to fill in an anamnestic survey (i.e., birth, breast and/or formula feeding habits, dummy- or thumb-sucking, headaches, dental and general trauma, pain in the muscles of the head and neck and nocturnal bruxism). The questionnaire pertaining to each child was then used at subsequent clinical examinations to notify the medical specialists of any related disorders, thereby assisting them in the formation of an accurate clinical picture. During this screening, each child underwent the following examinations: 1) dental/occlusal; 2) orthoptic; and 3) osteopathic-postural. These investigations were performed at the school by two dentists, one orthoptist and one osteopathist, under the expert supervision of a clinician specialized in School Medicine (from the ASL 3, 1^st^ District). The examination protocol adopted was based on previous studies [16,17].

Three clinical evaluation forms that were previously designed were used to record the dental, orthoptic and postural data, and a medical record of each child was compiled.

**Figure 1:**
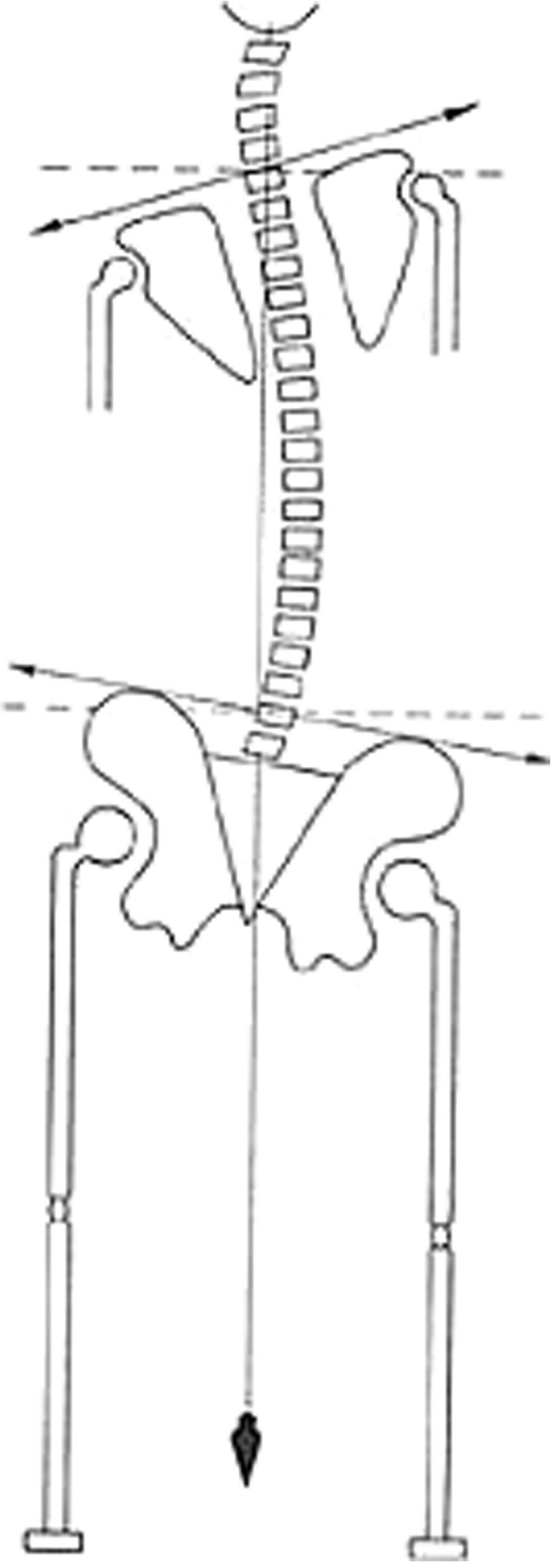
Postural assessment involved frontal and lateral inspection, investigation during trunk flexion and ambulation, and note of any asymmetry in the lower limbs or shoulders was taken.

**Figure 2:**
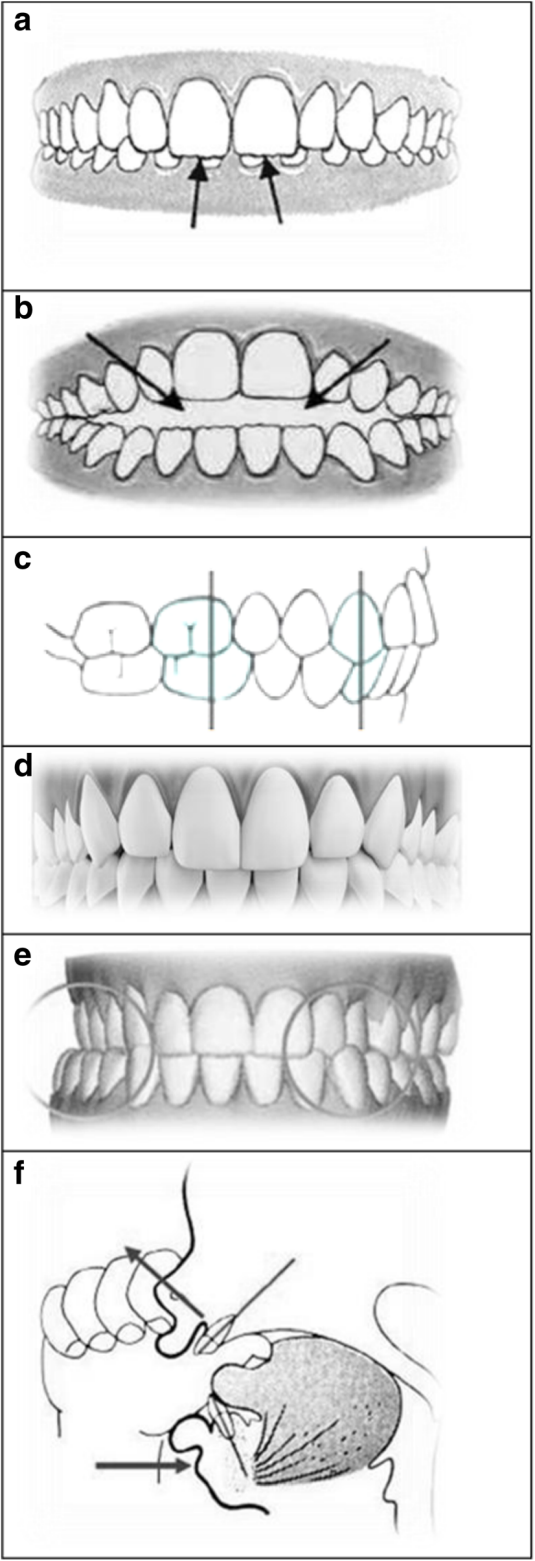
Occlusal alterations; (a) overbite alteration: deepbite; (b) overbite alteration: openbite; (c) molar and canine correct classification: class I relationship; (d) median line deviation; (e) alteration of the transversal relationship: crossbite; (f) bad habits: an example: sucking of finger.

**Figure 3:**
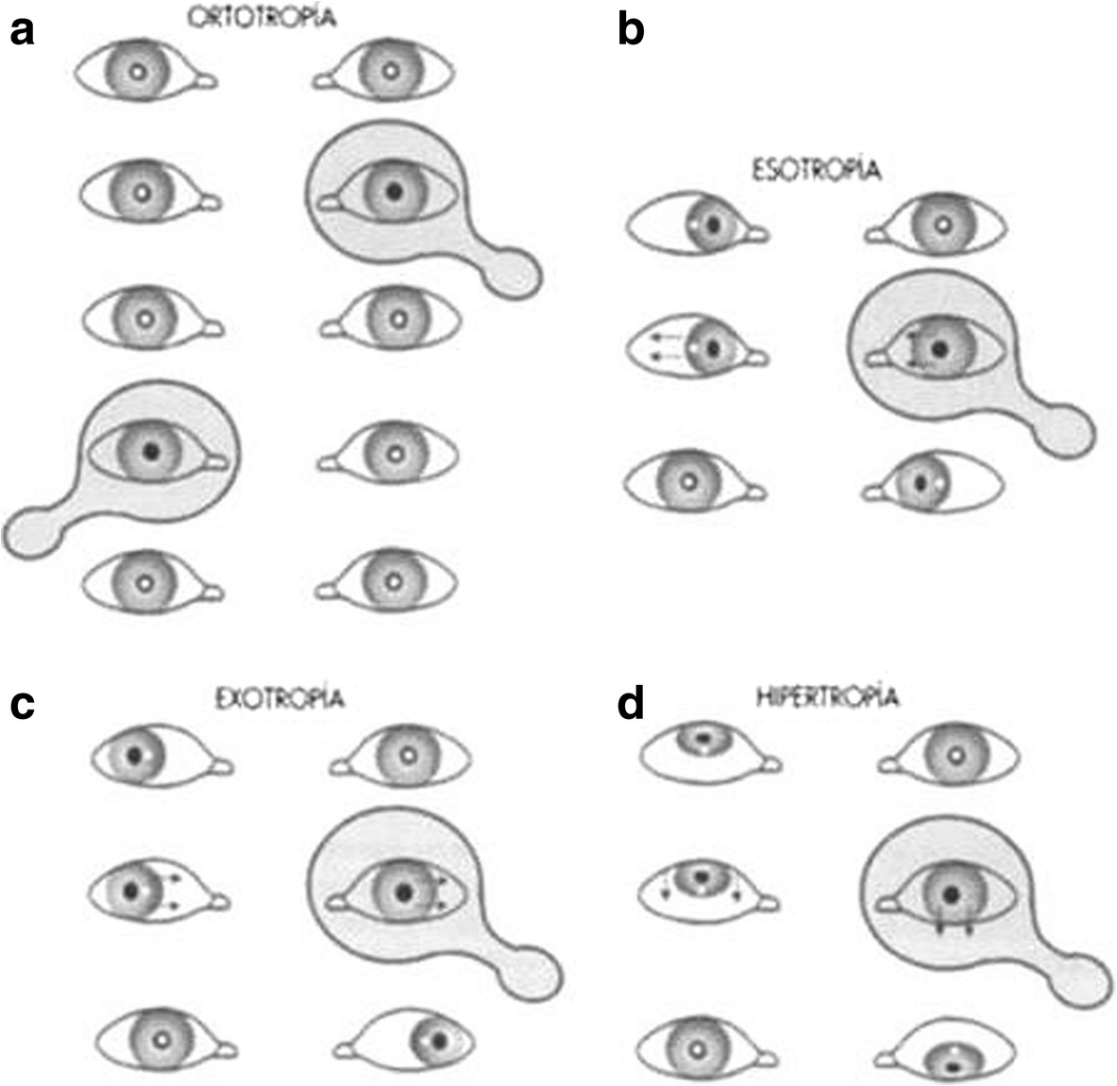
Cover test for eyes; (a) ortotrophia; (b) esotrophia; (c) exotrophia; (d) hipertrophia.

**Figure 4:**
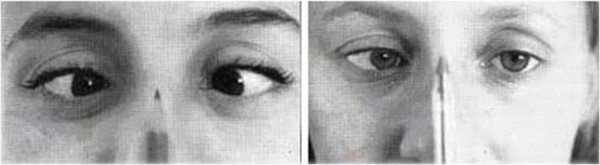
Convergence test.

**Figure 5:**
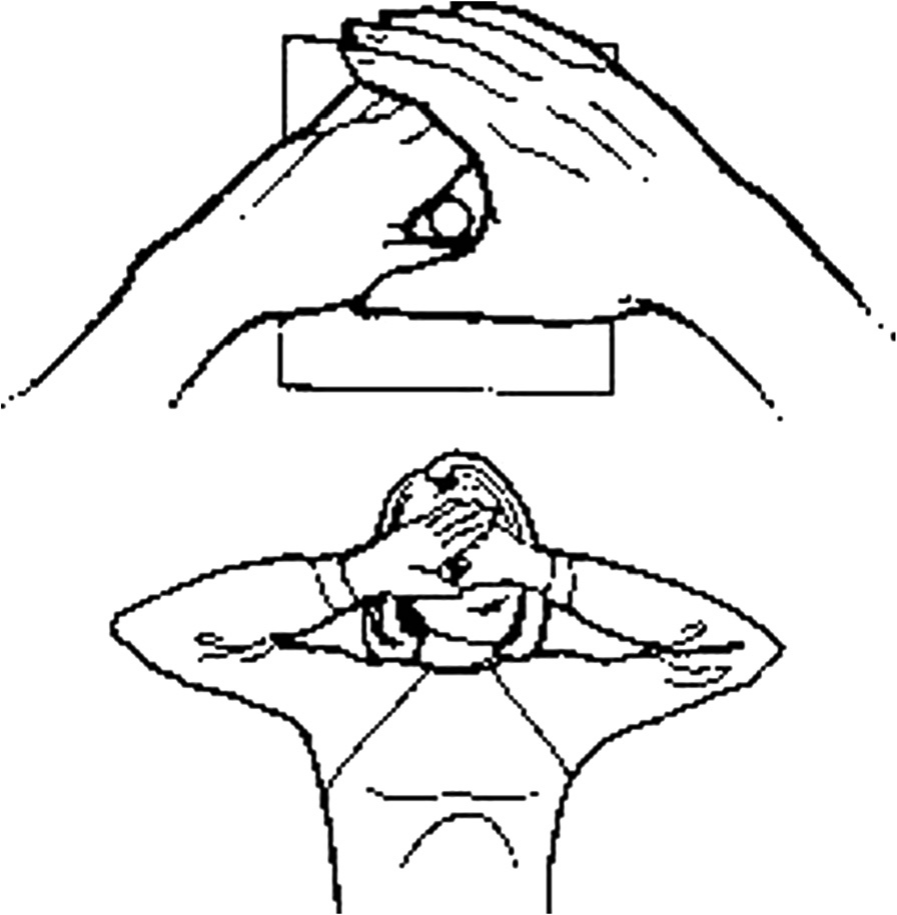
Dominant eye test.

Postural assessment involved frontal and lateral inspection, investigation during trunk flexion and ambulation, and note of any asymmetry in the lower limbs was taken (Figure 1). The collected dental information contained details of the molar and canine relationships, dental and facial midline, overbite, transversal relationships (cross-bite presence/absence), bad habits, hygiene and ongoing orthodontic treatment (Figure 2). The recorded orthoptic data included those pertaining to a cover test, convergence, ocular dominance and the Brock string test (Figures 3, 4, 5).

The following eye tests were carried out primarily to determine postural information:

Cover/uncover test (CT; Figure 3): The test was performed by covering and uncovering each eye in turn (e.g., with a hand or occluder) while the patient fixated on a small object. The purpose of the test was to determine any re-fixation movement in the uncovered eye. In this way, each eye was considered as a single entity and any axial defects (heterophoria) were detected by the presence of re-fixation movements in the uncovered eye (dissociation of the right and left eyes). An experienced ophthalmologist was then able to quantify the degree of phoria by passing a test card featuring increasing prism diopter values before the eyes until the re-fixation movement disappeared.

Convergence test (CT; Figure 4): The test was performed up to the base of the nose to determine the degree of tonic (dynamic), fusional and accommodative convergence.

There are two types of convergence:

Dynamic convergence (smooth pursuit system), which is considered pathological if the saccadic movements of one eye are slower than the other, the convergence movement of one or both eyes fails to complete, or the eye or eyes wander outwards when attempting to converge. Postural imbalance resulting from this condition will be greater if the defect is more pronounced in one eye.

Reflexive convergence (saccadic system), which seems to involve the saccadic rather than pursuit system, and is considered pathological if an eye fails to converge, stops in transit or wanders outwards when attempting to perform convergence. If the two eyes contradict each other, the convergence defect is considered to be present in both eyes. If the hypoconvergent eye protrudes and points downwards during reflexive convergence, a phoria or trophia is likely to be present.

Ocular dominance test (Figure 5): A card with a hole of diameter 2 cm is placed a distance of 10–15 cm from the patient’s eyes. The patient is then asked to stare through the hole at a distant object and the eye that automatically complies is the dominant eye. Confirmation of ocular dominance can be achieved by covering each eye in turn and repeating the test. Without moving the pierced card, the dominant eye will be the only one able to see the object in the center of the hole.

Parents were present and fully informed about the clinical findings. All patients approved the inclusion of their data in this study. The research reported in the paper was undertaken in compliance with the Helsinki Declaration. Ethical approval was granted by the Ethics Committee of the S. Martino Hospital- University of Genoa-National Institute for Cancer Research (P.R 21/12). Written consent was obtained after orientation to the study.

### Statistical evaluation

All data were analyzed in the form of percentages of subjects with a particular occlusal or postural or ocular situation. The percentages of subjects with postural or ocular diseases were compared among subjects with a normal, deep or open bite.

A chi-square test was used to evaluate the null hypothesis stating that the frequency distributions of gait posture, leg dysmetry or ocular diseases observed in our sample were consistent with a particular theoretical distribution. The null hypothesis was that the considered diseases were mutually exclusive and had the same probability for each type of teeth bite (open, normal or deep bite) because all the disease outcomes were equally likely to occur. The percentages were compared with a chi-square test. The p level was set at 0.05.

The software SPSS 9.0 was employed to evaluate statistical tests.

## Results

A total of 605 children students (mean age 8.5 ± 2.3 years; 45% males; 55% females) were examined.

Table 1 shows the correlation between gait and dental occlusion (open bite and deep bite).

**Table 1:** Distribution of physiological and pathological gait in our population

	**Normal bite (%)**	**Deep-bite (%)**	**Open-bite (%)**	**Total (%)**
**Physiological gait (%)**	86.92% (N = 226)	85.13% (N = 206)	85.30% (N = 87)	100% (N = 519)
**Pathological gait (%)**	13.08% (N = 34) (*)	14.87% (N = 36)	14.70% (N = 15)	100% (N = 85)
Total (%)	100% N = 260	100% (N = 242)	100% (N = 102)	N = 604

* significantly lower than the other two groups.

There was a prevalence of cases with an unphysiological gait in patients with an abnormal overjet (14.7%) or overbite (14.87%), while the percentage of patients with normal occlusion that showed an unphysiological gait was 13.08%.

In our sample, about 85.3%–86.9% of the subjects showed a physiological gait.

Table 2 shows the results for dysmetric legs. In our sample, the percentages of subjects with dysmetric legs were lower than the percentages of subjects with a pathological gait, suggesting that about 5%–7% of the cases with a pathological gait were due to other factors (Table 2). Also, about 93.85%–94.2% of the children had normal legs, without respect to the type of teeth bite (Table 2).

**Table 2:** Distribution of dysmetric legs in our population

	**Normal bite (%)**	**Deep-bite (%)**	**Open- bite (%)**	**Total (%)**
Dysmetric legs (%)	6.15% (N = 16)	5.76% (N = 14)	5.94% (N = 6)	100% (N = 36)
Normal legs (%)	93.85% (N = 244)	94.24% (N = 229)	94.06% (N = 95)	100% (N = 568)
Total (%)	100% N = 260	100% (N = 243)	100% (N = 101)	N = 604

* significantly lower than the other two groups.

Table 3 shows the data for the ocular dominant test. The majority of subjects had a right dominant eye (386 subjects vs. 221 subjects), which means 63%–67% had a right dominant eye and 33%–37% of the subjects had a left dominant eye. Subjects with an open bite showed a slightly different distribution with a lower difference between the percentages of right or left dominant eyes. In the ocular dominance test (Table 3), patients with an open bite with a right dominant eye vs. left dominant eye were 58.42% and 41.58%, respectively. Patients with a deep bite that showed a right dominant eye were 66.66% while 33.33% had a left dominant eye. Patients with normal occlusion with a right dominant eye were 62.7% and those with a left dominant eye were 37.3%.

**Table 3:** Distribution of dominant eyes data in our population

	**Normal bite (%)**	**Deep-bite (%)**	**Open-bite (%)**	**Total (%)**
Right dominant eye (%)	62.70% (N = 163)	66.66% (N = 164)	58.42% (N = 59)	100% (N = 386)
Left dominant eye (%)	37.30% (N = 97)	33.33% (N = 82) (*)	41.58% (N = 42) (*)	100% (N = 221)
Total (%)	100% (N = 260)	100% (N = 246)	100% (N = 101)	N = 607

* significantly lower than the other two groups.

Table 4 shows the results for ocular convergence diseases.

**Table 4:** Distribution of data about the ocular convergence diseases

	**Normal bite (%)**	**Deep-bite (%)**	**Open-bite (%)**	**Total (%)**
CT phoria	85.38% (N = 222)	83.53% (N = 203)	88.12% (N = 89)	100% (N = 514)
CTexophoria	8.84% (N = 23)	11.93% (N = 29) (*)	8.91% (N = 9)	100% (N = 61)
CT esophoria	4.23% (N = 11)	4.52% (N = 11)	2.97% (N = 3) (*)	100% (N = 25)
CT trophia	1.53% (N = 4)	0% (N = 0)	0% (N = 0)	100% (N = 4)
Total (%)	100% (N = 260)	100% (N = 246)	100% (N = 101)	N = 607

* significantly lower than the other two groups.

Subjects with an open bite showed a significantly lower percentage (2.97%) of CT-eso with respect to subjects with a normal bite or deep bite. Subjects with a deep bite showed a significantly higher percentage of CT-exo with respect to subjects with a normal or open bite.

Table 5 shows the data for ocular convergence (normal, pathological on the right side or pathological on the left side). Patients with an open bite showed a significantly higher prevalence (8.65%) of pathological convergence in the left eye with respect to subjects with a normal or deep bite. Patients with a deep bite showed a significantly lower percentage (3.70%) of pathological convergence in the right eye with respect to the other groups.

**Table 5:** Distribution of data about the ocular convergence

	**Normal bite (%)**	**Deep-bite (%)**	**Open-bite (%)**	**Total (%)**
Normal	89.18% (N = 231)	90.94% (N = 221)	86.54% (N = 90)	100% (N = 542)
Pathological in the right side	4.24% (N = 11)	3.70% (N = 9) (*)	4.80% (N = 5)	100% (N = 25)
Pathological in the left side	6.56% (N = 17)	5.34% (N = 13)	8.65% (N = 9) (*)	100% (N = 39)
Total (%)	100% (N = 259)	100% (N = 243)	100% (N = 104)	N = 606

* significantly lower than the other two groups.

## Discussion

### The dominant eye

A difference was noted in the observed percentages in the open bite children that showed a left dominant eye (41.58%), which was higher than children with a deep bite or normal bite. Deep bite subjects were more likely to show a right dominant eye (66.6%) with respect to children with a normal bite or open bite. The data suggested that the tendency of the vertical dimension of the bite (deep bite or open bite) was related to the dominant eye. The relationship between the dominant eye and the mandibular position was reported in terms of change in the transverse plane of the head posture after positioning eye patches over the dominant eye for 1 h [18].

### Ocular convergence disorders (phorias)

Subjects with an open bite showed a significantly lower percentage (2.97%) of CT-esophoria with respect to subjects with a normal bite or deep bite. As in other forms of strabismus, esodeviation can be controlled by fusional divergence (esophoria), intermittently controlled by fusional divergence (intermittent esotrophia) or constantly manifest (esotrophia). Subjects with a deep bite showed a significantly higher percentage of CT-exophoria with respect to subjects with a normal or open bite.

Other studies have shown the relationships of dental occlusion, the oculomotor system and visual stabilization. Evidence for a correlation between eyes and dental occlusion came from the use of mandibular orthopedic repositioning appliances, which simultaneously modify mandibular position and visual focusing tests using prismatic bars. These phenomena gradually disappear after removal of the appliance.

Associations also exist between TMD and oculomotor function. Some authors suggested a much higher prevalence of ocular convergence defects in TMD adults presenting with a limited maximal opening, myofascial pain and pain in the neck and shoulder area [18].

Numerous anatomical connections have been described between trigeminal systems and the nervous structures involved in maintaining posture. The mesencephalic nucleus of the trigeminus, which extends itself from the dorsal portion of the spinal trigeminal nucleus to the caudal part of the superior colliculus, is a sensorial nucleus with unique characteristics.

In the mesencephalic nucleus of the trigeminus, neurons associated with extraocular muscles are present along with the primary afferent neurons associated with the muscles of the MM, tooth pulp and periodontal ligaments. From the mesencephalic nucleus of the trigeminus, the neural pathways connect with the cerebellum, the reticular formation and the medial, inferior, lateral and superior vestibular nuclei.

This cross-sectional study was carried out by observing 605 subjects. Analysis of cross-sectional data usually consists of comparing the differences among the subjects, as well as yielding incidence rates for any single disorder. These data can only describe the incidence proportion of the contemporary presence of occlusal, orthoptic and postural disorders.

In this epidemiological survey, we assessed that:

About 13% of the children showed a pathological gait and, among them, patients with vertical anomalies of occlusion (deep bite or open bite) seemed to demonstrate a higher prevalence (with a statistically significant difference of 2%) of a pathological gait;

About 5%–6% of the entire sample demonstrated dysmetric legs, which can explain only 50% of the cases with a pathological gait;

The vertical dimension of occlusion revealed a slight relationship with the proper dominant eye, as deep bite patients showed a significantly lower tendency towards a left dominant eye (p < 0.05). Therefore, more were likely to show a right dominant eye. At the same time, deep bite subjects revealed a significantly lower frequency of pathological convergence in their right eye (p < 0.05). Deep bite subjects had the tendency to show more frequent CT exophoria at a significant level (p < 0.05), while open bite patients showed a significantly lower percentage of CT esophoria.

## Conclusions

In conclusion, postural, orthoptic and occlusal alterations may be clinically associated. Clinical connections or concomitant frequencies can be found among them, often with significant differences. From these data, it is not possible to state that there is a direct causal connection among them, but we may suppose that a causal connection may exist. Therefore, the treatment of such disorders requires the intervention of several specialists and a multidisciplinary approach. It is very important that pediatricians are aware of these possible clinical associations to direct young patients to the appropriate specialists that may treat these various disorders. In addition, it can be concluded that when a child is suffering from one of these disorders (postural, occlusal or orthotic), it may be useful for them to undergo screening for other possibly associated disorders.

## Competing interests

For this study, there was no conflict of interest in: (1) the study design; (2) the collection, analysis and interpretation of data; (3) the writing of the report; and (4) the decision to submit the paper for publication.

Armando Silvestrini-Biavati wrote the first draft of the manuscript and no honorarium, grant, or other form of payment was given to anyone to produce the manuscript.

## Authors’ contributions

ASB and PSB conceived the study; MM, EDM, ASB and PSB recorded the data; ST analyzed the data; ASB, MM, MS and PSB organized the manuscript and interpreted the results; MS and AP followed the statistical evaluation and reviewed the text; MS and ASB coordinated the work. All authors read and approved the final manuscript.

## Pre-publication history

The pre-publication history for this paper can be accessed here:

http://www.biomedcentral.com/1471-2431/13/12/prepub
